# An Overview of *Phytophthora* Species on Woody Plants in Sweden and Other Nordic Countries

**DOI:** 10.3390/microorganisms11051309

**Published:** 2023-05-17

**Authors:** Iryna Matsiakh, Audrius Menkis

**Affiliations:** 1 Southern Swedish Forest Research Centre, SLU Forest Damage Centre, Swedish University of Agricultural Sciences, Sundsvägen 3, 23422 Alnarp, Sweden; iryna.matsiakh@slu.se; 2 Institute of Forestry and Park Gardening, Ukrainian National Forestry University, Pryrodna 19, 79057 Lviv, Ukraine; 3 Department of Forest Mycology and Plant Pathology, Uppsala BioCenter, SLU Forest Damage Centre, Swedish University of Agricultural Sciences, P.O. Box 7026, 75007 Uppsala, Sweden

**Keywords:** forest, pathogens, oomycetes, tree diseases, woody plants

## Abstract

The genus *Phytophthora*, with 326 species in 12 phylogenetic clades currently known, includes many economically important pathogens of woody plants. Different *Phytophthora* species often possess a hemibiotrophic or necrotrophic lifestyle, have either a broad or narrow host range, can cause a variety of disease symptoms (root rot, damping-off, bleeding stem cankers, or blight of foliage), and occur in different growing environments (nurseries, urban and agricultural areas, or forests). Here, we summarize the available knowledge on the occurrence, host range, symptoms of damage, and aggressiveness of different *Phytophthora* species associated with woody plants in Nordic countries with a special emphasis on Sweden. We evaluate the potential risks of *Phytophthora* species to different woody plants in this geographical area and emphasize the increasing threats associated with continued introduction of invasive *Phytophthora* species.

## 1. Introduction

The genus *Phytophthora*, which includes fungus-like microorganisms, also known as water molds, belongs to the family Peronosporaceae and phylum Oomycota in the Stramenopila kingdom [1,2,3,4]. Initially, the classification of *Phytophthora* species was based on morphological characters (e.g., sporangia, homothallism, and configuration of antheridia), showing the presence of six groups [5]. However, homology and homoplasty among different *Phytophthora* species showed a high plasticity of the morphological features and their often inseparability [6,7,8,9]. Since the 2000s, the number of described *Phytophthora* species increased by over 180 species, which was primarily due to the use of novel molecular techniques, reaching a total of 326 species distributed in 12 phylogenetic clades [10]. Consequently, the taxonomy of the genus *Phytophthora* shifted from the morphology-based methods towards the development of molecular markers for multilocus phylogenies [11,12,13,14,15]. For example, phylogenies of *Phytophthora* species were constructed using an internal transcribed spacer (ITS) region [11], four nuclear and mitochondrial genes [16], or seven nuclear markers [17]. A more recent study advanced the *Phytophthora* phylogeny by including more than 180 species and by creating ancestral phylogeny reconstructions on the sporangial papillation [18]. These studies allow researchers to better understand the evolution of the genus *Phytophthora* and to link molecular phylogenies and individual morphological and physiological traits.

There are several techniques available to detect and identify *Phytophthora* species. A classical detection method is based on the cultivation of *Phytophthora* species on different nutrient media favoring the development of morphological features, which can be used in taxonomical classifications. Several molecular methods have also been developed to study *Phytophthora* species and/or subspecies. The methods used for the identification of isolates to the species level include the DNA sequence analysis of a specific locus or of several specific loci, such as the ITS region, 60S ribosomal protein L10, β-tubulin, enolase, HS protein 90, large subunit rRNA, TigA gene fusion, translation elongation factor 1α, *cox1*, *cox2*, *nad1*, *nad9*, *rps10*, or *secY* [16,17,19,20,21,22,23,24]. The methods used in population biology studies of *Phytophthora* species include restriction fragment length polymorphism (RFLP), amplified fragment length polymorphism (AFLP), random amplified polymorphic DNA (RAPD), microsatellites, single nucleotide polymorphisms (SNPs), or mitochondrial haplotype analyses [20,21,25,26,27,28,29,30,31,32,33,34,35,36,37,38,39,40]. The enzyme-linked immunosorbent assay (ELISA) includes *Phytophthora*-specific kits for high throughput use in multi-well plates and ImmunoStrips or lateral flow devices (LFD) for single-use or for on-site diagnostics [41,42,43,44,45,46,47,48,49]. Conventional PCR techniques can also be highly specific for the detection of the pathogen in a small amount of plant tissue (e.g., using Scorpion primers) [50]. Loop mediated isothermal amplification (LAMP) is another technique (based on four primers annealing to different regions of the target organism), which can be used for diagnostic purposes (e.g., for the rapid detection of *P. ramorum*) [50,51]. A genus-specific diagnostic multiplexed marker, which is based on the mitochondrially encoded *cox1* and *cox2* spacer regions, can also be used for the detection of regulated *Phytophthora* species [52].

Most *Phytophthora* species are considered primary plant pathogens with a hemibiotrophic or necrotrophic lifestyle, however, the genus also includes the aquatic species, saprophytes and opportunistic necrotrophic pathogens [3,4,6,53,54,55,56,57,58]. Many *Phytophthora* species are known to cause various disease symptoms in different tree species, shrubs, and crops across the world, infecting plants from seedlings (annual crops) to mature trees [3,55,59,60,61,62]. They can cause root rot, damping-off of plant seedlings, aerial bleeding cankers on stems, and blight of foliage, buds, or fruits in different plant families [55,63,64,65,66,67,68]. Generalist species, such as *P. cinnamomi*, *P. cryptogea*, *P. nicotianae*, *P. palmivora*, and *P. ramorum*, can infect many woody plant hosts, whereas specialist species, such as *P.* × *alni*, *P. lateralis*, and *P. quercina*, are associated with a limited number of host species [3,69].

The genus *Phytophthora* is considered one of the most serious biosecurity threats to forest ecosystems worldwide. Several tree disease outbreaks caused by *Phytophthora* species (e.g., *P. ramorum*, *P. kernoviae*, *P lateralis*, *P. pseudosyringae*, and *P. austrocedri*) have been reported, resulting in a significant mortality of multiple woody plant hosts [66,70,71,72,73,74]. For example, *P. cinnamomi* caused the mortality of *Eucalyptus marginata* in Jarrah ecosystems in Australia, *P. lateralis* was a causal agent of *Chamaecyparis lawsoniana* dieback in North America, and the hybrid *P.* × *alni* was shown to cause mortality, dieback, and root and collar rot of *Alnus* spp. in Europe [3,75,76,77].

Invasive *Phytophthora* species are also increasingly emerging as the most damaging non-native tree pathogens [78,79,80]. For example, intensive sampling of forest soils and streams revealed that several *Phytophthora* species were invasive, causing severe damage to new non-coevolved host plants [81]. Among these, species such as *P. plurivora* [82], *P. pini* [83], *P. multivora* [84], *P. mengei* [85], *P. capensis* [86], *P. elongata* [87], and *P. acerina* [88] are considered to be pathogenic to a number of woody plants.

Recent studies have also revealed a high diversity of *Phytophthora* species in different growing environments (e.g., nurseries, urban and agricultural areas, and forests) [3,10,89,90,91,92,93,94,95,96,97]. The spread of *Phytophthora* is often linked to global plant trade, as well as unintentional human introductions (e.g., with shoes or tools) [93,98,99,100,101]. The assessment showed that 92% of the stock of commercial plants in over 700 forest- and ornamental nurseries in 23 European countries was infected by *Phytophthora* species, including the most aggressive ones with the potential to be introduced to new natural and semi-natural environments [89]. This also highlights a high risk of *Phytophthora* introduction to urban areas via infected ornamental woody plants.

Despite the available knowledge of *Phytophthora* species in northern Europe, their impact on woody plants in different environments is still uncertain and requires further attention, especially since such impact can be affected by different abiotic factors, possibly impeding the establishment of some *Phytophthora* species [102,103]. Additionally, the primacy of the *Phytophthora* species as causal agents of woody plant diseases might often be misinterpreted due to their specific lifecycles, which require specific detection methods, and the time of sampling, as inoculum levels often depend on the phase of the disease [3].

In this review, we summarize the available knowledge on *Phytophthora* species associated with woody plants in Sweden and other Nordic countries; the threat that they may pose in different environments; and their origin, host range, symptoms of damage, pathways of introduction, and geographic spread. Using this information, we evaluate the potential risks associated with *Phytophthora* species to different woody plants under Nordic conditions and demonstrate that their presence poses a potential threat for further spread within and between neighboring countries. We also would like to raise awareness about the risks associated with the continued introduction of invasive *Phytophthora* species via imported plant materials and other means. Despite the management strategies of forest pathogens in Nordic countries, disease control efforts can be hampered, owing to a lack of knowledge on how serious the threat by *Phytophthora* species may be to local woody plants in different environments.

## 2. Searching for Information

The occurrence of *Phytophthora* species in Sweden and in other Nordic countries and their current status was identified by searching both existing public databases and publications included in the Web of Science and Mendeley databases. All the databases were accessed between October 2022 and February 2023. We confined our searches to *Phytophthora* reports in the Web of Science and Mendeley using different combinations of keywords “*Phytophthora*”, “Sweden”, “Norway”, “Denmark”, “Finland”, and “oomycetes”. Grey literature was limited to published information about *Phytophthora* spp. in Sweden. The following public sources were used to extract country-level distribution data for *Phytophthora* species: Forest Phythophtoras of the World; IDphy: molecular and morphological identification of *Phytophthora* based on the types; Invasive Species Compendium CABI; EPPO Global Database; SLU Skogskada; identification of common *Phytophthora* species using a Lucid Key; Phytophthora Research Center. The SLU Artdatabanken (2021) was used to assess the risk of invasiveness of *Phytophthora* species in Sweden (https://doi.org/10.15468/j43wfc) (accessed on 24 February 2023).

Only *Phytophthora* species associated with woody plants in Sweden were considered, and results of this analysis are summarized in Table 1.

Based on analyses of the available sources, 17 *Phytophthora* species derived from nurseries, natural and anthropogenic forests, urban areas, and rivers/water sources in Sweden. These species include the following: *Phytophthora alni* species complex (*P. alni* subsp. *alni*, *P. alni* subsp. *uniformis*, *P. alni* subsp. *multiformis*), *P. cactorum*, *P. cambivora*, *P. cinnamomi*, *P. citrophthora*, *P. cryptogea*, *P. gonapodyides*, *P. quercina*, *P. lacustris*, *P. ramorum*, *P. megasperma*, *P. pini*, *P. plurivora*, *P. pseudosyringae*, *P. rosacearum*, *P. syringae*, and *Elongisporangium undulatum* (syn. *P. undulata*) (Table 1). According to the data on invasive taxa in Sweden (SLU Artdatabanken, 2021), only nine *Phytophthora* species (*P*. × *alni*, *P. cactorum*, *P. cambivora*, *P. cinnamomi*, *P. cryptogea*, *P. gonapodyides*, *P. quercina*, *P. ramorum*, and *P. plurivora*) are considered invasive in Sweden, while the status of the remaining species is uncertain, despite being present in the country.

In Sweden, six *Phytophthora* species *(P.* × *alni*, *P. cactorum*, *P. cambivora*, *P. gonapodyides*, *P. plurivora*, and *P. quercina)* were found to be associated with different types of damage on woody plants, and nine *Phytophthora* species (*P. cinnamomi*, *P. citrophthora*, *P. cryptogea*, *P. ramorum*, *P. pini*, *P. pseudosyringae*, *P. rosacearum*, *P. syringae*, and *Elongisporangium undulatum*) were detected on woody ornamentals. *P.* × *alni*, *P. lacustris*, *P. plurivora*, and *P. megasperma* were discovered in rivers/streams or other water sources.

During the period of 1954 to 1958, a number of fungi that occurred at a high frequency on dying seedlings were isolated mainly from diseased seedlings from forest nurseries in central Sweden. *Phytophthora cactorum* was isolated from forest tree seedlings together with other pathogens *Fusarium orthoceras*, *F. solani*, *F. culmorum*, *F. oxysporum*, *Pythium intermedium*, and *P. debaryanum.* The first report about the detection of *Phytophthora* in Sweden was in 1961 [108]. The period between 1999 and 2006 was devoted to research on oak decline in Sweden, including studies on *Phytophthora* [110,118,120]. Since 2010, damage caused by *Phytophthora* has been discovered in recreational forests, parks, and urban settings around the Skåne county [109,121]. In 2012, quarantined species *P. ramorum* was reported several times on imported *Rhododendron* sp. [122], however, there are no reports of *P. ramorum* infecting trees in Sweden. More recently (2016–2018), extensive studies on *Phytophthora* were conducted across Sweden to evaluate the distribution, risk, and potential threat that new invasive *Phytophthora* species may pose to Swedish forests, cities, and landscapes [105,123]. Several *Phytophthora* species were also reported from Christmas tree plantations [113]. The occurrence of *Phytophthora* species on woody plants in Denmark, Finland, and Norway is shown in Table 2.

## 3. Characteristics of *Phytophthora* Species Detected on Woody Plants in Sweden and Other Nordic Countries

***Phytophthora* × *alni*** Clade 7a

**Key woody hosts:** *Alnus alnobetula*, *A. glutinosa*, *A. cordata*, *A. incana*, *A. rubra*, *A. rubra* subs. *tenufolia*, *Castanea sativa.*

**Symptoms:** canker, collar rot, and dieback of alders

**Aggressiveness:** *Phytophthora* disease of alder is now widespread in Europe in the riparian ecosystems where alder commonly grows. In Europe, surveys and modeling show that the risk of infection is higher in warmer, slow-moving waters, and in fine-textured soils, especially clay loams. Although the disease is usually observed along river systems, it has been found in sites far from riverbanks or other water courses, e.g., in orchard shelter belts and in new woodland plantings. This suggests that alder trees were already infected prior to planting [3]. *P.* × *alni* is an aggressive pathogen as, e.g., inoculations of mycelia culture on one-year-old seedlings of *A. glutinosa* and *B. pendula* showed the development of lesions in 89% and 67% of seedlings, respectively [124].

**Table 2:** Occurrence of *Phytophthora* species on woody plants in Denmark, Finland, and Norway.

Species	Country	Hosts	Source
*Phytophthora alni* subsp. *uniformis* ^a^	Denmark	*Alnus glutinosa*	[125]
	Finland	*A. glutinosa*	[124]
*P. cactorum*	Denmark	*Betula pendula*	[126]
	Finland	*A. glutinosa*, *B. pendula*, *Rhododendron* sp.	[127,128,129]
*P. cambivora*	Norway	*Abies procera*, *Fagus sylvatica*	[130,131]
*P. citrophthora*	Norway	*Chamaecyparis lawsoniana*	[132]
*P. inundata*	Norway	*Abies nordmanniana*	[132]
*P. megasperma*	Norway	*Abies lasiocarpa*	[132]
*P. pini*	Finland	*Rhododendron* sp.	[129]
	Norway	*Chamaecyparis lawsoniana*	[132]
*P. plurivora* ^b^	Denmark	*A. glutinosa*, *Fraxinus excelsior*	[125,133,134]
	Finland	*Rhododendron* sp., *Syringa vulgaris*	[129,135]
	Norway	*F. sylvatica*	[131]
*P. ramorum*	Norway	*Rhododendron catawbiense*, *Pieris japonica*, *Viburnum fragrans*	[136]
	Finland	*Rhododendron* sp.	[137]

^a^ Previously *Phytophthora uniformis*; ^b^ Previously belonged to *P. citricola* complex.

**Figure 1:**
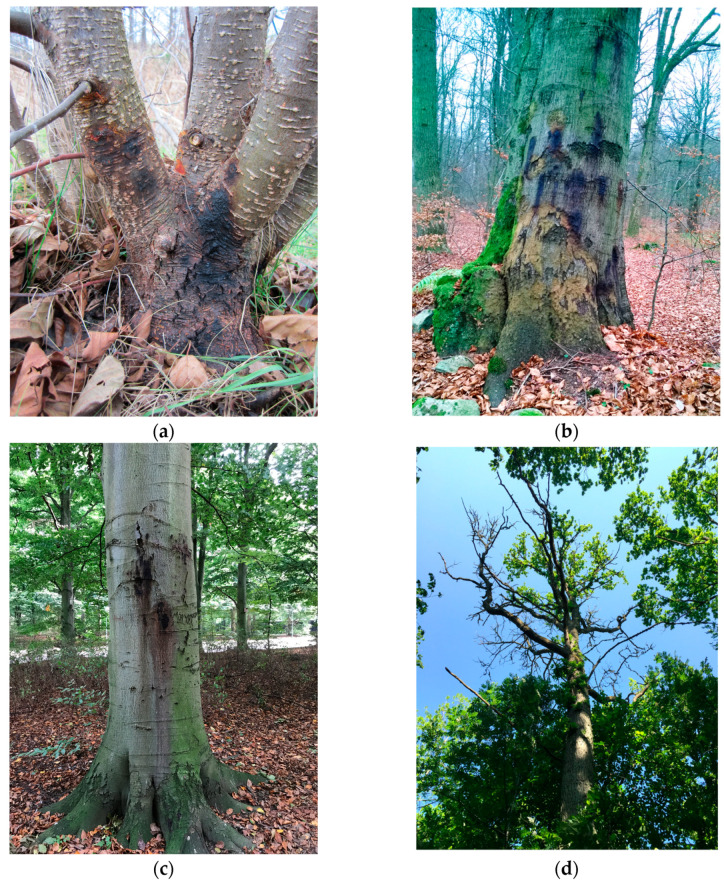
*Phytophthora* infected trees in Sweden: (**a**) *Phytophthora alni* lesions at the stem base of *Alnus glutinosa* (photo by Miguel Angel Redondo; retrieved from https://internt.slu.se/en/news-originals/2020/2/alders-lack-resistance-against-aggressive-type-of-pathogen/; accessed on 21 March 2023); (**b**) infected *Fagus sylvatica* with characteristic “bleeding” lesions on the stem in Kullaberg nature reserve (photo by Michelle Cleary); (**c**) infected *Fagus sylvatica* with “bleeding” lesions on the stem caused by *P. plurivora* and *P. gonapodyides* in Pildamms Park, Malmö city (photo by Mimmi Blomquist); (**d**) extensive crown dieback of *Quesrcus robur* in Visingrö oak forest caused by *P. plurivora* root rot infection (photo by Michelle Cleary).

**Occurrence:** In Sweden, the first report of the *P. alni* complex was from nurseries and alder-planted areas in the southwest in the 1990s [104]. In 2006 and 2010, it was discovered on *A. incana* at the Klarälven river in the city of Karlstad. A comprehensive study on the *Phytophthora alni* complex in Sweden was carried out between 2013 and 2018 to investigate the pathways of introduction and the spread of the two subspecies, *alni* and *uniformis* [103,138]. Both species were associated with *Phytophthora* bleeding cankers on 93% of declining *A. glutinosa* and some *A. incana* trees along the riverbanks, and within sampling plots connected to river swamps or ponds (Figure 1a) [103,138]. It was considered that both *P. alni* subsp. *alni* and *P. alni* subsp. *uniformis* are invasive species that arrived in Sweden with plant material imported to forest nurseries, and that these species may further spread into natural ecosystems. *P. alni* subsp. *uniformis* was widespread throughout the country, whereas *P. alni* subsp. *alni* was only discovered in the southern and coastal part of Sweden [103,138]. *P. alni* subsp. *alni* is one of the most aggressive *Phytophthora* species [139], however, it is more sensitive to cold winters [140]. It was concluded that southern Sweden could be the northernmost distribution limit of *P.* × *alni*, however, there is a possible risk of its migration northwards due to climate change [138,141]. Due to the poor genetic potential of alder trees to resist *P alni* subsp. *alni*, alder decline is expected to increase in Sweden in the future [123]. In Finland, *P. alni* subsp. *uniformis* (identified as *Phytophthora* cf. *uniformis*) was found, for the first time, to cause dark stem lesions on *A. glutinosa* seedlings in 2015 [124]. In Denmark, *P. alni* subsp. *uniformis* (identified as *Phytophthora uniformis*) was isolated for the first time from symptomatic trees of *A. glutinosa* in 2016 [125].

***Phytophthora cactorum*** Clade 1a

**Key woody hosts:** *Abies* sp., *Acer* sp., *Aesculus hippocastanum*, *Fagus sylvatica*, *Juglans regia*, *Fraxinus excelsior*, *Malus domestica*, *Populus alba*, *Quercus* sp., *Rhododendron* sp. (however, in total, at least 154 genera of vascular plants in 54 families are affected).

**Symptoms:** root, collar, and crown rot on many species; brown-reddish stem lesions; slow decline or rapid dieback, depending on age and location of infections; root rot of nursery plants. For example, on woody plants, such as *Malus domestica*, *P. cactorum* is causing crown and root rot; on *Betula* spp., it causes stem lesions; and on rhododendron, it causes root rot and dieback symptoms [142].

**Aggressiveness:** *P. cactorum* is unequivocally a serious pathogen of a wide range of plant species. Despite its broad geographic distribution and host range, *P. cactorum* has similar symptomatology with other species of *Phytophthora.* Therefore, it is difficult to ascribe specific damage to *P. cactorum* and evaluate the extent of its damage to forest trees. There are several reports of noticeable “outbreaks” on *Fagus* and *Betula* [143], however, it appears that there is a considerable host specificity among strains of this pathogen. Swedish isolates of *P. cactorum* together with *P. cambivora* and *P. plurivora* were used for inoculation of common conifer and broadleaf tree species in Sweden (*Pinus sylvestris*, *Picea abies*, *Larix* × *eurolepis*, *Betula pendula*, *Quercus robur*, *Fagus sylvatica*, *Populus trichocarpa*, and *Tilia cordata*) to determine their relative susceptibility to root pathogens [144]. All the tested *Phytophthora* species caused stem lesions of varying lengths on different host trees, except for species in the Pinaceae family, which had low susceptibility to the tested *Phytophthora* spp. Two-year-old bare-root seedlings of *B. pendula*, *Q. robur*, *F. sylvatica*, and *P. sylvestris* appeared to be susceptible to *P. cactorum* infection [144]. Inoculation trials in Finland using a Danish isolate, which was isolated from *B. pendula* in 2009, revealed that *P. cactorum* caused relatively small lesions on *Rhododendron* sp. and *P. abies*, moderate lesions on *B. pendula*, and no infections on *Q. robur* or *P. sylvestris* [129]. In an in vitro study using two-month-old *B. pendula*, roots inoculated with *P. cactorum* often showed dark discolorations, loss of fine roots, and decreased branching [126], even though discolorations are not a specific symptom of *Phytophthora* infections [127]. The symptoms of the aboveground parts included reduced height growth, lower chlorophyll fluorescence, significantly longer dark or brown discolorations in the stems, and a higher proportion of brownish and wilting leaves [126]. Furthermore, inoculation trials on three-month-old *B. pendula* and *A. glutinosa* seedlings showed that *P. cactorum* was able to kill 40% of the *B. pendula* seedlings, but caused only small lesions on 40% of *A. glutinosa* seedlings [128]. However, in the inoculation trials, *P. cactorum* was found to cause low-to-moderate symptoms on *Rhododendron* sp. and *P. abies*, and no symptoms on *P. sylvestris* [129]. *P. cactorum* was also able to cause lesions on non-wounded *B. pendula* seedlings [145]. In stem inoculation trials, Orlikowski et al. [134] showed that *A. glutinosa*, *B. pendula*, and *Prunus padus* were highly susceptible to *P. cactorum*. *Acer saccharinum*, *Corylus avellana*, *Q. robur*, *Rubus caesius*, *Sorbus aucuparia*, and *Tilia cordata* were moderately susceptible, while *Sambucus nigra* and *Sorbus aucuparia* were not susceptible. Interestingly, *P. cactorum* can be detected in *B. pendula* seven years after outplanting, however, at this stage, the effect of stem lesions on seedling mortality or on the number of leader shoots is limited [146]. Bunyaviruses were shown to significantly reduce hyphal growth and the production of sporangia and their size, but not the pathogenicity of *P. cactorum* [147].

**Occurrence:** In Sweden, *P. cactorum* was first recognized as one of the root parasites causing damping-off disease in forest nurseries in 1961 [108]. Since the beginning of the 1990s, the oak population has been declining in Sweden [148], and in 2003, *P. cactorum* was found to be associated with oak decline in southern Sweden [110]. Later, *P. cactorum* was isolated from the diseased *F. sylvatica* in the city of Malmö and Stora Köpinge municipality in 2016 [109]. Recently, *P. cactorum* was detected on *F. sylvatica* and *Q. robur* in forest nurseries (close to the rivers Säveån, Kävlingeå, and Ätran) (composing 26.3% of all detected *Phytophthora* spp.), in urban *F. sylvatica* forests (near the river Lagan) (15%), and in natural forests affecting *F. sylvatica* (near the river Ronnebyån), *A. alba*, and *P. abies* (near the river Ätran) (30%) [105]. In Finland, *P. cactorum* was found for the first time in necrotic stem lesions of *B. pendula* seedlings in forest nurseries in 1991 [127] and in stem lesions of *A. glutinosa* seedlings in 1995 [128]. It was also detected on symptomatic *Rhododendron* sp. seedlings during surveys that were carried out between 2004 and 2010 [129]. Irrigation water used in Finnish forest nurseries was shown to be the possible source of *P. cactorum* inoculum [149]. The Finnish isolates of *P. cactorum* were shown to have a 17.5 °C optimal growth temperature [135], i.e., much lower than the 25−30 °C reported in other studies [150,151], and that these isolates survived −5 °C temperatures on agar medium for up to 14 days [135]. The observations above suggest that Nordic isolates of *P. cactorum* can be better adapted to local conditions and, in the future, may pose a threat to *B. pendula* seedlings in forest nurseries and reforestations.

***Phytophthora cambivora*** Clade 7a

**Key woody hosts:** *Abies alba*, *Acer platanoides*, *Aesculus hippocastanum*, *Alnus glutinosa*, *Castanea denatata*, *C. crenata*, *C. sativa*, *Fagus sylvatica*, *Quesrcus robur*, *Taxus brevifolia*, *Platanus orientalis*, *Juglans regia*, *Malus domestica*, *Rhododendron* sp., *Pieris* sp., *Prunus* sp., *Ulmus* sp.

**Symptoms:** canker, collar and root rot, bleeding cankers

**Aggressiveness:** *P. cambivora* is an invasive pathogen that survives and spreads in different environments. Its ability to survive as a saprotroph in the soil and to produce oospores (resting structures) increases its invasiveness. It was described as a causal agent of ink disease on chestnut trees [152,153,154]. The infection causes root destruction, which leads to leaf chlorosis and wilting in the canopies. Depending on environmental conditions, the disease may lead to a quick or to a progressive dieback of infected trees [152,155]. Inoculation tests on *Abies* seedlings also showed the ability of *P. cambivora* to infect and cause characteristic canker symptoms [130].

**Occurrence:** In Sweden, the first detection of *P. cambivora* was in association with oak health deterioration [110]. It was found together with *P. cactorum* in soil samples in one of ten surveyed forest stands. Later, *P. cambivora* was detected in soil samples collected near *F. sylvatica* trees with bleeding stem cankers in Bokskogen near the city of Malmö (Figure 1b) [109]. Nowadays, *P. cambivora* is present in nurseries, urban areas, and natural forests, and is mainly associated with *F. sylvatica* decline and bleeding cankers on the stems [105]. Inoculation of the stems showed that the Swedish isolate of *P. cambivora* is highly pathogenic to *F. sylvatica*, *B. pendula*, *Tilia cordata*, *Q. robur*, and *Populus trichocarpa. P. trichocorpa* is a non-native tree species in Sweden but it is important for biomass production. Therefore, the establishment of new *P. trichocarpa* plantations for energy should take place using clones more tolerant to *Phytophthora* infections [144]. In Norway, *P. cambivora* was detected for the first time on a 15-year-old *Abies procera* in 2004 [130]. The symptoms included cankers on the stems up to 1.5 m above the ground and dieback of the basal branches. There were 25% of trees that were already dead or dying. Infections of *P. cambivora* on *F. sylvatica* were observed for the first time in 2011, resulting in bleeding cankers [131]. The infected trees were in the areas of Larvik and Ås, which represent the northern limit of *F. sylvatica* distribution, showing that a northern location is not a limiting factor for the spread and infection by *P. cambivora*. The infected trees had a circumference between 40 and 310 cm, and the majority of the cankers were at the height of 0.1−2 m above the ground. Additional symptoms included crown dieback, chlorotic foliage, epicormic shoots, and cracked bark. The infection frequency in some areas around Larvik was up to 4.9% in 2012, but it was up to 9.2% in Ås in 2014 [131]. In addition to *P. cambivora*, *P. plurivora* and *P. gonapodyides* were detected in the water near the diseased trees in Larvik, and both species proved to be pathogenic on *F. sylvativa*. Today, *P. cambivora* can be considered an established species in different environments. To limit its spread, monitoring should take place in nurseries and on seedlings used for outplanting [81].

***Phytophthora cinnamomi*** Clade 7a

**Key woody hosts:** *Abies* sp., *Castanea sativa*, *Quercus* sp., *Chamaecyparis lawsoniana* (266 genera in 90 families; commonly hardwood trees, including more than 1000 species [55]).

**Symptoms**: root rot, heart rot, wilt; causes ink disease of chestnut in conjunction with *Phytophthora cambivora.*

**Aggressiveness:** It is currently the most important *Phytophthora* pathogen of forest trees, and it is also destructive to woody ornamentals, especially rhododendrons and other Ericaceae, and orchard crops, including avocado. It is now widespread, owing to the international trade of plants, and continues to be destructive in the forests of Australia, Mediterranean countries, Mexico, and the SE United States, and is of increasing concern in the forests and wildlands of western North America. With the changing climate, *P. cinnamomi* is expected to expand its range and cause more damage, particularly in Europe and North America.

**Occurrence:** In Sweden, *P. cinnamomi* was detected in the rhizosphere soil of *Rhododendron luteum* ‘Whitethroat’ and *Stewartia pseudocamellia* growing in the same nursery, thereby representing the first record of this pathogen in a commercial stock of ornamental plants in the country [112].

***Phytophthora citrophthora*** Clade 2a

**Key woody hosts:** *Citrus* sp., *Aesculus hippocastanum*, *Buxus* sp., *Castanea sativa*, *Chamaecyparis lawsoniana*, *Juglans regia*, *Rhododendron* sp. (in total, 88 genera in 51 families).

**Symptoms:** root rot, stem necrosis, canker, fruit rot, twig blight, seedling blight

**Aggressiveness:** *P. citrophthora* causes brown rot disease of citrus and is an economically important pathogen of citrus crops. It can also cause a dieback of rhododendron and other ornamental plants.

**Occurrence:** In Sweden, *P. citrophthora* was found in a nursery of *Rhododendron catawbiense* in 2018 [105]. In Norway, it was detected on *Chamaecyparis lawsoniana* [132].

***Phytophthora cryptogea*** Clade 8a

**Key woody hosts:** *Abies concolor*, *A. fraseri*, *A. procera*, *Chamaecyparis* sp., *Cupressus* sp., *Juglans regia*, *Malus domestica*, *Pinus mugo*, *P. nigra*, *P. contorta*, *Pseudotsuga menziesii*, *Rhododendron catawbiense*, *R. maximum* (in total, 141 genera in 49 families).

**Symptoms:** damping-off, foot rot, stem rot, leaf rot, wilt.

**Aggressiveness:** *P. cryptogea* is primarily a soil-borne plant pathogen in the temperate regions, but it also exists in nature (fresh water) as a saprotroph. It is most active at temperatures between 10 °C and 20 °C [55]. It is a serious plant pathogen in many countries, causing great damage to ornamentals produced in nurseries, greenhouses, and hydroponics. *P. cryptogea* is an aggressive soil-borne pathogen of fir species, which are produced as Christmas trees [156,157].

**Occurrence:** In Sweden, *P. cryptogea* was detected in soil samples associated with symptomatic *F. sylvatica* trees in a nursery (near the river Ätran) and in an urban forest (near the river Alsterån) [105]. It was also found in soil samples in Christmas tree plantations and, together with *P. megasperma*, demonstrated a high aggressiveness to *P. abies* and *A. nordmanniana* trees [113].

***Phytophthora gonapodyides*** Clade 6b

**Key woody hosts:** *Chamaecyparis lawsoniana*, *Corylus avellana*, *Fagus sylvatica*, *Juglans regia*, *Malus domestica*, *Quercus* sp., *Rhododendron* sp.

**Symptoms:** stem bleeding cankers, root rot

**Aggressiveness:** *P. gonapodyides* is considered as a weak parasite with saprophytic abilities usually associated with aquatic environments, such as rivers, riparian areas, and wetlands [55]. However, some isolates of *P. gonapodyides* can be highly virulent [134,158]. Their aggressiveness appears to be stimulated by prolonged root flooding and cool soil conditions. *P. gonapodyides* can hinder seed germination and cause root rot and stem lesions in *Q. robur* and *Q. ilex* [159,160].

**Occurrence:** In Sweden, the first report of *P. gonapodyides* was in 2016, when the pathogen was isolated from characteristic bleeding cankers on *F. sylvatica* trees growing in Pildamms Park in the city of Malmö (Figure 1c) [114]. It was suggested that recent changes in local climatic conditions, such as high summer precipitation coupled with mild winter temperatures, could favor the multicyclic spread of *P. gonapodyides* via zoospores and/or that the increased average age of *F. sylvatica* stands contributed to their higher susceptibility [114]. *P. gonapodyides* was also reported in a nursery (Lagan area) in association with *F. sylvatica* seedlings [105]. In Denmark, *P. gonapodyides* was recovered from rainwater ponding in an old declining *F. excelsior* stand [134].

***Phytophthora inundata*** Clade 6a

**Key woody hosts:** *Aesculus hippocastanum*, *Olea* sp., *Salix* sp., *Vitis* sp.

**Symptoms:** root and collar rot of trees or shrubs in wet or flooded areas

**Aggressiveness:** *P. inundata* is responsible for wilting and the root rot of olive trees [161]. It can also act as an opportunistic, albeit aggressive root pathogen. On *A. nordmanniana*, it caused poorly developed roots and brown to reddish discoloration under the bark at the stem base and downwards. The foliage exhibited drought symptoms, with leaves that were pale green, yellow, or brown [132].

**Occurrence:** There are no reports from Sweden. In Norway, *P. inundata* and *P. megasperma* were reported from Christmas tree plantations of *A. nordmanniana* and *A. lasiocarpa* in 2004, respectively [132]. Approx. 70% of *A. nordmanniana* and 25% of *A. lasiocarpa* were symptomatic. As the site was grassland for decades with no history of Christmas tree cultivation, it was suggested that the disease followed imported transplants [132].

***Phytophthora megasperma*** Clade 6b

**Key woody hosts:** *Aesculus hippocastanum*, *Castanea sativa*, *Juglans regia*, *Prunus domestica*, *Pseudotsuga menziesii*, *Sorbus aucuparia*

**Symptoms:** root rot, crown rot, storage rot, seedling damping-off, fruit rot, foot rot, stem canker, tuber rot, collar rot, sudden wilt, apoplexy, stunting, chlorosis. The symptoms of *P. megasperma* on *A. lasiocarpa* were pale yellow foliage and girdling at the stem bases [132].

**Aggressiveness:** It is primarily a root-rotting organism, causing the most serious losses on fruit and broadleaf trees. It appears to be restricted to more temperate regions of the world, however, its oospores can survive for up to 5 years, either free in the soil or in host tissue [162]. Prolonged wet conditions and heavy clay soils and soil impaction layers, which allow the maintenance of high soil water content, are often needed for the development of disease epidemics by *P. megasperma* [163].

**Occurrence:** In Sweden, *P. megasperma* was isolated from roots of a symptomatic *P. abies* seedling [113]. In Norway, *P. megasperma* was reported in association with *A. lasiocarpa* [132]. Both species *P. cryptogea* and *P. megasperma* may become problematic for Christmas tree and bough production, especially in saturated soils, which favor disease development.

***Phytophthora pini*** Clade 2c

**Key woody hosts:** Pinaceae

**Symptoms:** root rot, canker

**Aggressiveness:** There is only limited information about this species. It can cause root rot and rapid mortality of olive trees [164]. Inoculations of *P. abies* seedlings using both wound-mycelia and zoospore suspension showed that after seven days, *P. pini* caused 100% disease incidence and a high frequency of severe symptoms [135].

**Occurrence:** In Sweden, *P. pini* was reported in commercial nurseries (near rivers of Säveån and Mölndalsån) in association with *R. catawbiense* [105]. In Finland, it was also detected on *Rhododendron* sp. [129].

***Phytophthora plurivora*** Clade 2c

**Key woody hosts:** *Acer platanoides*, *Aesculus hippocastanum*, *Alnus glutinosa*, *Fraxinus excelsior*, *Quercus robur*, *Quercus petrea*, *Tilia cordata*, *Fagus sylvatica*, *Rhododendron* sp.

**Symptoms:** stem cankers, collar and root rot, dieback. *P. plurivora* can cause wilting and discoloration of current year shoots (on *P. abies* seedlings), bark necroses, fine root losses, and dieback on at least 45 woody host species [129,135].

**Aggressiveness:** *P. plurivora* is a highly aggressive plant pathogen, which has a worldwide distribution and a high diversity of hosts. *P. plurivora* is a hemibiotrophic organism that possesses the ability to infect living tissues and to continue its life cycle on dead tissues. In inoculation trials, *P. plurivora* was able to cause relatively large lesions or, in many cases, stem girdling on *Rhododendron* sp., *B. pendula*, *A. incana*, *A. glutinosa*, and *P. abies*, showing a high virulence on several woody plants and especially on *P. abies* as compared to other *Phytophthora* spp. tested [129]. The disease incidence on *P. abies* was also shown to be dependent on a particular isolate and inoculation method, as there were 83.3−100% disease incidence using wound inoculation with living mycelia and 0−77.8% using zoospores [135]. In *P. abies* shoot tissues, *P. plurivora* can grow both inter- and intracellularly, which is largely in the vascular tissues [135]. *Pinus sylvestris* and *Q. robur* showed no symptoms after four weeks of *P. plurivora* inoculation [129,137]; however, in other studies, this pathogen caused extensive bark lesions on *Q. robur* after a longer time [165]. In stem inoculation trials, Orlikowski et al. [134] showed that *A. saccharinum*, *A. glutinosa*, *B. pendula*, *C. avellana*, *P. padus*, *R. caesius*, and *S. nigra* were highly susceptible to *P. plurivora*, while *Q. robur*, *S. aucuparia*, and *T. cordata* were moderately susceptible. In addition to stem inoculations, soil infestation trials may also be needed to examine the susceptibility of fine roots of different tree species to *P. plurivora* and other *Phytophthora* spp. [129].

**Occurrence:** In Sweden, the earliest report of *P. plurivora* was from alder trees in Asslebyn (Bengtsfors locality) in Sept 2012 [105], even though the species was likely found during surveys near the city of Nyköping [103]. In 2016, *P. plurivora* was detected in soil samples and bleeding stem lesions of *F. sylvatica* in the city of Malmö (Figure 1c) [109]. It is one of the most abundantly detected *Phytophthora* species in natural forests and urban areas with declining and symptomatic *F. sylvatica* and *Q. robur* trees (Figure 1d) [105]. *P. plurivora* was also found to be highly virulent on *F. sylvatica* and *Q. robur* seedlings, causing large lesions, thus, it should be considered as a high-risk species to Swedish forests with a potential to severely destabilize the broadleaf forest ecosystems [114]. *P. plurivora* was also reported from Denmark and Norway as a disease agent of several deciduous tree species [125,133,166]. In Norway, *P. plurivora* was reported on *F. sylvatica* in a park in Oslo and in Ålesund [131]. Interestingly, the infection process for some *F. sylvatica* trees was rather fast and took only two years before the tree was dead. In Finland, surveys in 2005 on symptomatic *Rhododendron* sp. resulted in the detection of *P. plurivora* (originally identified as *P. inflata*) [129,137].

***Phytophthora pseudosyringae*** Clade 3a

**Key woody hosts:** *Quercus* spp., *Fagus sylvatica*, *Alnus glutinosa*, *Carpinus betulus*

**Symptoms:** root and collar rot, stem bleeding cankers

**Aggressiveness:** It is an aggressive pathogen on several broadleaf tree species.

**Occurrence:** In Sweden, *P. pseudosyringae* was reported causing basal cankers and dieback on horse chestnut in June 2014 in Sankt Jörgens Park in the city of Gothenburg [117].

***Phytophthora quercina*** Clade 3b

**Key woody hosts:** *Quercus* spp.

**Symptoms:** rot of fine roots, overall oak decline

**Aggressiveness:** *P. quercina* is often associated with other *Phytophthora* spp. [148], shows adaptation to different site conditions and soil pH, and has a high host specificity i.e., a high aggressiveness to different oak species [150,160]. *P. quercina* is also well-adapted to temporary dry conditions, possibly due to its particularly thick oospore walls [150]. Oaks with *P. quercina* or other *Phytophthora* spp. in their rhizosphere have ca. 50% higher probability of exhibiting severe aboveground disease symptoms than oaks without *Phytophthora* spp. [167]. Jönsson et al. [118] showed that Swedish isolates of *P. quercina* had the capacity to induce fine-root dieback of *Q. robur* seedlings growing in acid, N-rich but otherwise nutrient-poor forest soils (dominant in Sweden), as well as in high pH, nutrient-rich soils under the mesic water regime. Their aggressiveness, together with a high infection rate (all the seedlings were infected) showed a potential capacity of *P. quercina* to infect plants in acid forest soils [118]. In addition, the stress-induced susceptibility of the seedlings and/or increased aggressiveness of the pathogen in the forest soil could be factors accounting for differences of root dieback between soil types [118].

**Occurrence:** The decline of European oaks mainly occurs in trees older than 100 years, and in this process, trees may survive for a long time. It is only under exceptional circumstances that oaks may die in large areas [160,168]. A similar decline of oaks (in particular, *Q. robur*) has occurred in Sweden during the recent decades [148]. The reason for this loss was unclear until the three different *Phytophthora* species were recovered from 11 out of 32 oak stands in the southernmost part of the country, with *P. quercina* being the most frequent species [110]. However, a weak association was found between the occurrence of *P. quercina* and the vitality of mature oak stands [169]. Thus, the decline of oaks in southern Sweden can probably be attributed to several different site-specific factors, such as infection by *P. quercina* or unusual weather events, which interact with a number of biotic and abiotic factors, leading to oak decline [170]. Later, Jönsson-Belyazio and Rosengren [171] summarized that *P. quercina* contributes to oak decline in southern Sweden. A conceptual model for the development of *Phytophthora* disease in *Q. robur* suggested that the link between the root damage caused by *Phytophthora* species and overall tree vitality is in the assimilation and allocation of carbon within the plants [120]. More recently, *P. quercina* was also found in both urban (Mölndalsån area) and natural forests (Säveån and Lyckebyån areas), but not in forest nurseries [105].

***Phytophthora ramorum*** Clade 8c

**Key woody hosts:** *Abies* sp., *Aesculus hippocastanum*, *Alnus* sp., *Betula pendula*, *Fagus sylvatica*, *Fraxinus excelsior*, *Larix kaempferi*, *Notholithocarpus densiflorus*, *Pseudotsuga menziesii*, *Quercus* sp., *Rhododendron ponticum*, *Rhododendron* sp.

**Symptoms:** lethal stem cankers, shoot dieback, foliage blight

**Aggressiveness:** *P. ramorum* is one of the most aggressive *Phytophthora* species. It is considered a highly invasive species due to its ability to spread, persist, and reproduce in new environments. The pathogen can infect plants in nurseries situated in close proximity to streams, later causing significant outbreaks on outplanted ornamentals. Spread events appear to be associated with either the movement of infected plant parts, normally from large wild infestations, or the introduction of infected plants, normally from infested ornamental nursery stock [172]. Infected nursery plants, such as *Rhododendron*, *Camellia*, and *Viburnum*, often contribute to long distance dispersal of the pathogen. Inoculation trials on stems showed that in addition to *Rhododendron* sp., *P. ramorum* caused necrotic lesions on *A. glutinosa*, *A. incana*, and *B. pendula*, while *P. sylvestris* and *P. abies* showed no disease symptoms [129,137], even though the pathogen might be able to infect individual *P. abies* needles [173]. Inoculation trials suggest that the damage can be substantial, as used isolates of *P. ramorum* were able to cause stem lesions in over 80% of *B. pendula* and over 30% of *A. glutinosa* seedlings [129].

**Occurrence:** In Sweden, the first report of *P. ramorum* was in 2002, which was found on 11 plants of *Rhododendron* sp. [174]. In 2017, it was detected on two plants of *Rhododendron yakushimanum* in a nursery in the municipality of Skurup. In 2018, *P. ramorum* was detected on four *Rhododendron* plants in a private garden in the municipality of Klippan. In Finland, *P. ramorum* was detected for the first time in 2004 [137]. It was found on marketed plants of *Rhododendron* spp., which were imported from other EU member states. The same year, *P. ramorum* was also discovered on *Rhododendron* sp. in a Finnish nursery, and detection was also successful in the following years, i.e., 2004−2010, except 2007, showing the persistent establishment of this pathogen despite the annual sanitation measures [129]. In Norway, *P. ramorum* was reported for the first time in 2002 [136]. It was isolated from symptomatic *R. catawbiense* in a nursery in Bergen. In the following years, the number of locations with *P. ramorum* has gradually increased (in 2004, there were 29 new locations, and in 2005, there were 43 locations), showing a broader distribution and/or rapid spread within the country. Apart from rhododendron, *P. ramorum* was also detected on *Pieris japonica* and *Viburnum fragrans*, and the latter was heavily infected. *P. ramorum* was most likely imported to Norway with symptomless plants and/or with plants that had mild symptoms, which are difficult to detect using random controls [136].

***Phytophthora rosacearum*** Clade 6a

**Key woody hosts:** *Malus domestica*, *Prunus* spp. (Rosaceae)

**Symptoms:** pathogenic

**Aggressiveness:** Not clear

**Occurrence:** In Sweden, *P. rosacearum* was reported in commercial nurseries (near Kävlingeå) in association with *Prunus laurocerasus* [105].

***Phytophthora syringae*** Clade 8d

**Key woody hosts:** *Aesculus hippocastanum*, *Fagus sylvatica*, *Camelia* sp., *Rhododendron* sp., *Prunus* sp. (29 genera in 14 families, including *Syringa vulgaris* (Oleaceae) and Rosaceae).

**Symptoms:** twig blight, fruit rot, root and collar rot, stem canker, wilt, leaf spot, and shoot dieback of lilac

**Aggressiveness:** *P. syringae* is known to infect nursery plants, particularly apple and pear trees. It infects plants through wounded areas and is most pathogenic during cold and wet weather conditions.

**Occurrence:** In Sweden, *P. syringae* was found in the soil in the vicinity of horse chestnut growing in Pildamms Park in the city of Malmö [109]. It was also detected in a nursery (near Kävlingeå) and in urban forests (near the Nyköpingsån river) in association with *R. catawbiense* [105].

## 4. *Phytophthora* Species Detected in the Water and Soil in Sweden

Two species, *P. alni* subsp. *alni* and *P. alni* subsp. *uniformis*, were found in the Säveån and Mölndalsån rivers near Gothenburg in 1996 and 1998 [104]. Systematic sampling of the *Phytophthora* species was performed in 16 rivers in southern Sweden in 2013 using inspection of active bleeding cankers on alder trees along the rivers [103]. *P. alni* subsp. *multiformis* was detected in 7 rivers and *P. alni* subsp. *uniformis* was in 13 rivers, while *P. plurivora* was isolated from alders growing along three rivers [103]. *Phytophthora cryptogea*, *P. gonapodyides*, *P. lacustris*, *P. megasperma*, *P. plurivora*, *P. taxon paludosa*, and an unknown *Phytophthora* species were isolated from waterways and soil samples in Christmas tree plantations in southern Sweden [113]. Recently, *P. scandivavica*, a new waterborne species, was isolated from the riverbank soil in the Kiruna area in northern Sweden [175].

## 5. Concerns Regarding *Phytophthora* Pathogens on Woody Plants

Over the last 60 years and since the calamity of *P. cinnamomi* in Western Australia and Victoria in the 1960s, incidences of *Phytophthora* diseases have exponentially increased globally [3,176,177]. In Nordic countries, the majority of *Phytophthora* detections took place after the 2000s, highlighting the importance of these destructive pathogens, and providing critically needed information on their occurrence, spread, and possible impact in different environments. However, the concerns remain that new *Phytophthora* species may arrive and establish itself, which should be tackled by harmonizing both phytosanitary regulations/processes and exchanging information between EU countries [178,179,180,181,182]. Most of the *Phytophthora* species detected in Nordic countries are exotic invasive pathogens, often introduced through the ‘plants-for-planting’ pathway. Global change poses a risk for the spread and establishment of these *Phytophthora* species in natural ecosystems, which may subsequently result in the decline of local forest tree species [89,183,184]. To reduce such damage in Nordic countries, targeted efforts and knowledge are needed to monitor new and manage existing *Phytophthora* species of woody plants.

Assessing the potential risks of *Phytophthora* spp. to woody plants (Table 3) is a highly complex task that is further complicated by the ability of different *Phytophthora* species to cause multiple infections on different hosts and to produce hybrids, which might be more virulent than their progenitors under specific conditions, thereby facilitating adaptation of these *Phytophthora* species to different environments [55,185,186,187]. Due to their multi-cycling nature and a strong dependence on free water in the soil, *Phytophthora* species can also predispose woody plants to other abiotic and biotic factors by primarily colonizing fine roots and weakening their hosts [55]. For disease management, it is important to understand which *Phytophthora* species can be pathogenic and cause disease in a particular host. The presence of aggressive *Phytophthora* species in Sweden and other Nordic countries (e.g., *P.* × *alni*, *P. cactorum*, *P. cambivora*, *P. gonapodyides*, *P. plurivora*, or *P. quercina*) means that these should be considered as a serious threat to woody plants in urban and forest ecosystems (Table 3). In Sweden, more than half of the territory is covered by forests, which are mainly composed of coniferous tree species; however, due to a mild climate, broadleaved tree stands dominate in the southern part of the country. In these stands, *F. sylvatica*, *Quercus* sp., *F. excelsior*, *B. pendula*, *U. glabra*, and *A. glutinosa* are the dominant and ecologically most important tree species that are planted both for forestry purposes and as ornamentals in urban and landscape settings. These tree species are also increasingly planted to promote both biodiversity and sustainable land use and enhance the resilience of local forests to climate change [188]. However, there is a rising concern regarding increasing damage caused by *Phytophthora* species on broadleaved tree species, especially *F. sylvatica*, *A. hippocastanum*, *A. glutinosa*, *A. incana*, and *Q. robur* [105,109,115,117]. This represents a new challenge to broadleaved forestry, as, according to the Swedish Forestry Act [189], selected valuable broadleaved tree species must be used for the regeneration of broadleaved forests.

Climate change should also be considered, as it can escalate *Phytophthora* damage by altering the susceptibility of woody hosts and modifying the development of *Phytophthora* pathogens [190,191,192]. For example, damage caused by *P.* × *alni*, *P. cinnamomi*, *P. ramorum*, *P. pseudosyringae*, or *P. plurivora* are considered to increase due to climate change [191]. Warmer winters may not only increase the activity of *P.* × *alni*, but may also lead to a northern expansion of its range [141], while warmer summers and droughts are likely to favor *Phytophthora* species adapted to higher temperatures [191]. In Sweden, climate change modelling predicts an increase of the annual temperature between 3 °C and 7 °C, and the increase of local precipitation up to 40% [193]. Similarly, 3 °C to 5 °C higher winter and summer temperatures, and up to a 25% longer annual rainfall period are expected in the future in southern Sweden [194]. The Swedish Meteorological and Hydrological Institute (SMHI) has already recorded droughts during the summer of 2018 and high temperatures during the summer of 2022, including a temperature record of 37.2 °C on 21 July 2022, which was the highest temperature in Sweden since 29 July 1947 [195]. These climatic changes may have a substantial effect on the activity and spread of *Phytophthora* species in the future.

Another concern is related to the ‘never-ending story’ about the presence of *Phytophthora* species in the nurseries. In Sweden, the early reports of *Phytophthora* causing damage to woody plants came from nurseries [108]. The richness of the *Phytophthora* species detected in nurseries, anthropogenic forests, or urban greeneries is higher than in natural forests, demonstrating the potential introduction of these pathogens with infected nursery plants [89,93,109,138]. Indeed, nurseries, commercial ones in particular, are well-known to be an important pathway of introduction of invasive pathogens globally, including *Phytophthora* species in Europe [78,89,196,197,198]. Despite chemical treatments, which usually do not cure the disease, infected nursery plants often arrive visually symptomless, passing unnoticed through phytosanitary controls, thereby acting as ‘pathogen reservoirs’ and assuring the unintentional distribution of *Phytophthora* species [89,199,200,201]. The detection of *P. ramorum* on ornamental woody plants in several Nordic countries (Finland, Norway, and Sweden; Table 1 and Table 2) is one of the examples of accidental introduction of *Phytophthora* pathogens to new areas [136]. The detection of *P. cinnamomi*, which is one of the most aggressive invasive plant pathogens, on imported plant material in Sweden highlights the importance of surveillance activities for the early detection of *Phytophthora* pathogens and the provision of relevant knowledge for plant growers.

In Nordic countries, there is also a concern about the increasing risk of *Phytophthora* damage to Christmas tree plantations. Denmark is one of the largest exporters of Christmas trees to many European countries, including Sweden (6%) and Norway (3%) [202]. Although there are no reports about *Phytophthora* in Christmas trees plantations in Denmark, reports from Norway [132] and Sweden [113,115] show that *Phytophthora* may become a threat to Christmas tree production in the future. Indeed, *Phytophthora* species were introduced with imported Christmas tree seedlings to Norway, repeatedly showing the risk of possible future problems, including hybridization, in local forest nurseries and/or Christmas tree plantations [203].

## 6. Management of *Phytophthora* Diseases

One of the best approaches to *Phytophthora* management is the prevention of its introduction and establishment. Once *Phytophthora* spp. have been introduced, they will remain for many years due to the longevity of their resting propagules. However, the presence of *Phytophthora* infection does not necessarily mean that the tree will die, as some trees may successfully prevent continued growth of the pathogen around the trunk, at least for some time [204]. Nevertheless, management measures are often recommended to limit *Phytophthora* infections and the spread of these pathogens.

Some general management guidelines include restricting the deposition of organic waste in the vicinity of areas (forests, parks, or city plantations) sensitive to *Phytophthora* infestations and on infested sites eliminating human, animal, and vehicle movement; establishing routines for the thorough cleaning of machines and equipment used in management operations; harvesting dead/infected woody plants when the ground is frozen, which also helps to prevent soil compaction by used machines; prohibiting the use of materials (e.g., woodchips) from diseased woody plants in nature; improving root aeration by draining wet and especially flooded areas; and, if possible, adding organic mulch on top of the roots of woody plants [131,204,205]. The use of chemical fungicides and/or biological control products is another important management measure in highly managed environments, such as nurseries. For the establishment of new plantations, it is essential to use healthy nursery stock that has been thoroughly examined for the presence of *Phytophthora*. The replacement of susceptible woody plant species by those that are more tolerant to *Phytophthora* infections and are well-adapted to environmental conditions of a specific planting site can be another management measure. For example, to reduce the incidences of *Phytophthora* spp. in Christmas tree plantations, it is recommended to plant spruce instead of fir [206]. Invasive pests, such as *Arion vulgaris*, which may be vectors for the transmission of *Phytophthora* spp. to woody plants, should also be controlled [207].

## 7. Conclusions

Since the first report on *Phytophthora* species on woody plants in Sweden, the number of detected *Phytophthora* species and the number of disease incidences has sharply increased both in Sweden and in other Nordic countries. The introduction of new *Phytophthora* species with imported plants and/or by other means, recent changes in local climatic conditions, and the availability of new detection methods appears to be among the major reasons for this increase. Field observations and inoculation trials demonstrate that some of the detected *Phytophthora* species can be aggressive pathogens, posing a high risk to several woody plants in Nordic countries. To prevent and/or reduce damages, targeted efforts and knowledge are needed to monitor new and manage existing *Phytophthora* species of woody plants.

## Figures and Tables

**Table 1:** Occurrence of *Phytophthora* species associated with woody plants in Sweden.

Species Fungorum Current Name	EPPO Status in the Country	EPPO Species Status	First Report of Species in Sweden	Current Status in Sweden *	Geographical Distribution	Infected Plant Host in Sweden	Site of Species Detection	References
*Phytophthora alni* species complex: hybrid *P.* × *alni* and its two parental subspecies, *P.* × *uniformis* and *P.* × *multiformis*	EU regulated non-quarantine pest (RNQP)	Invasive, alien	1990s (1996-98)	*P. alni* subsp. *alni*—invasive, present	Säveån and Mölndalsån river systems along the west coast near Gothenburg; Ljungbyhed; Klarälven; rivers in southern Sweden	*Alnus glutinosa*, *A. incana*	Rivers, nurseries	[103,104,105,106,107]
			1990s (1996-98)	*P. alni* subsp. *uniformis* –invasive, present	Säveån and Mölndalsån river systems along the west coast near Gothenburg; Ljungbyhed; Klarälven; rivers in southern Sweden			
			2006	*P. alni* subsp. *mltiformis*—introduced, present, localized	Rivers in southern Sweden			
*Phytophthora cactorum* (Lebert and Cohn) J. Schröt.	EU regulated non-quarantine pest (RNQP) (Annex IV)	Invasive, saprophytes	1961, 2003, 2016	Invasive, present, uncertain	Säveån, Kävlingeå, Lagan, Ätran, Ronnebyån, oak forest stands in southern Sweden, Malmö	*Alnus glutinosa*, *Fagus sylvatica*, *Quercus robur*, *Abies alba*, *Picea abies*	Natural forests, nursery, urban areas (parks)	[105,108,109,110]
*Phytophthora cambivora* (Petri) Buisman, Meded. Inst.	EU regulated non-quarantine pest (RNQP)	Invasive	2003	Invasive, introduced, present	Southern Sweden (sites close to Malmö and Kalmar)	*Quesrcus* robur L., *Fagus sylvatica* L., *Aesculus hippocastanum* L	Natural forests, rivers, nurseries, urban areas (parks)	[105,109,110,111]
*Phytophthora cinnamomi* Rands, Meded. Inst.	EU Regulated Non-Quarantine Pest’ (RNQP) (Annex IV)	Invasive	2017	Invasive, present, uncertain	Southern Sweden	Water and rhizosphere soil of *Rhododendron luteum* ‘Whitethroat’, *Stewartia pseudocamellia*	Commercial nursery	[112]
*Phytophthora citrophthora* (R.E. Sm. & E.H. Sm.) Leonian	EU regulated non-quarantine pest (RNQP) (Annex IV)	Invasive	2018	Uncertain, present, restricted	Säveån	*Rhododendron catawbiense*	Nursery	[105]
*Phytophthora cryptogea* Pethybr. & Laff.	EU regulated non-quarantine pest (RNQP)	Invasive	2019	Invasive, present, widespread	Southern Sweden (Ätran), Västra Götaland, Halland, Skåne, Blekinge, Kalmar	*Alnus glutinosa*, *Fagus sylvatica*, *Quercus robur*	Anthropogenic forest, nursery, waterways, and soil samples in Christmas tree fields	[105,113]
*Phytophthora gonapodyides* (H.E. Petersen) Buisman	Non-EU EPPO status	Invasive, saprophytes	2016	Invasive, present, restricted	Southern Sweden (Pildammsparken in Malmö, Lagan)	*Fagus sylvatica*	Urban areas (parks), nursery, waterways, and soil samples in Christmas tree fields	[105,113,114]
*Phytophthora lacustris* Brasier, Cacciola, Nechw., T. Jung and Bakonyi	Non-EU EPPO status	Uncertain	2017	Uncertain, present, restricted	Southern Sweden (Västra Götaland, Halland, Skåne, Blekinge, Kalmar)	Waterways and soil samples in Christmas tree fields	Nursery	[113]
*Phtophthora megasperma* Drechsler, J. Wash.	Non-EU EPPO status	Uncertain	2015	Present, uncertain, restricted	Southern Sweden (Västra Götaland, Halland, Skåne, Blekinge, Kalmar)	*Picea abies* waterways and soil samples in Christmas tree fields	Nursery	[113,115,116]
*Phytophthora pini* Leonian	Non-EU EPPO status	Uncertain	2020	Uncertain, present, restricted	Säveån, Mölndalsån	*Rhododendron catawbiense*	Nursery	[105]
*Phytophthora plurivora* T. Jung and T.I. Burgess	Non-EU EPPO status	Risk of invasiveness	2016	Invasive, present, restricted	Southern Sweden (Västra Götaland, Halland, Skåne, Blekinge, Kalmar), Säveån, Ätran, Lagan Kävlingeå	*Betula pendula*, *Fagus sylvatica*, *Quercus robur*, waterways and soil samples in Christmas tree fields, *Rhododendron* sp.	Anthropogenic forest, natural forests, nursery, urban areas	[105,109,112,113]
*Phytophthora pseudosyringae* T. Jung and Delatour, in Jung, Nechwatal, Cooke, Hartmann, Blaschke, Osswald, Duncan and Delatour	Non-EU EPPO status	Uncertain	2014	Uncertain, present, restricted	Gothenburg	*Aesculus hippocastanum*	Urban areas	[117]
*Phytophthora quercina* T. Jung	Non-EU EPPO status	Risk of invasiveness	2003	Invasive, present; restricted distribution (widespread in the south)	Mölndalsån, Lyckebyån, Säveån, oak forest stands in southern Sweden	*Quesrcus robur*	Anthropogenic forest, natural forests	[105,118]
*Phytophthora ramorum* Werres, De Cock and Man in ‘t Veld, in Werres, Marwitz, Man in ‘t Veld, De Cock, Bonants, De Weerdt, Themann, Ilieva and Baayen	EU emergency measures (formerly), PZ quarantine pest (Annex III)	Invasive	2002, 2017	Invasive, absent, eradicated	Klippan, Skurup, Mölndalsån	*Rhododendron* sp., *Rhododendron catawbiense*	Urban areas (private gardens), nursery	[105,119]
*Phytophthora rosacearum* E.M. Hansen and W.F. Wilcox	Non-EU EPPO status	Uncertain	2020	Uncertain, present, restricted	Kävlingeå	*Prunus laurocerasus*	Nursery	[105]
*Phytophthora syringae* (Kleb.) Kleb.	Non-EU EPPO status	Uncertain	2020	Uncertain, present, restricted	Malmö, Nyköpingsån, Kävlingeå	*Aesculum hippocastanum*, *Rhododendron catawbiense*	Urban areas (soil in the parks), anthropogenic forest	[105,109]
*Elongisporangium undulatum* (H.E. Petersen) Uzuhasi, Tojo and Kakish. (syn. *Phytophthora undulata*)	Non-EU EPPO status	Uncertain	2020	Uncertain, present, restricted	Southern Sweden	*Rhododendron* × ‘Nova zembla, *Rhododendron hanceanum* × *R.* *keiskei*	Nursery	[112]

* SLU Artdatabanken (2021) was used to assess the risk of invasiveness of *Phytophthora* species in Sweden (Risk assessment of invasive taxa in Sweden. Version 1.6. https://doi.org/10.15468/j43wfc accessed via GBIF.org accessed on 24 February 2023), and the EPPO Global Database (https://gd.eppo.int/) (accessed on 24 February 2023) was used to verify pest status declared by Swedish NPPO.

**Table 3:** Potential risks of *Phytophthora* spp. to woody plants in Sweden.

Species	Risk of Damage to Tree Genus/Species		
	Low	Intermediate	High
***Phytophthora*** × ***alni***			*Alnus* sp.
***Phytophthora cactorum***	*Juglans regia*, *Malus domestica*, *Populus alba*	*Abies* sp., *Acer* sp., *Aesculus hippocastanum*, *Rhododendron* sp.	*Betula* sp., *Quercus* sp., *Fagus sylvatica*, *Fraxinus excelsior*
***Phytophthora cambivora***	*Taxus brevifolia*, *Platanus orientalis*, *Juglans regia*, *Malus domestica*, *Ulmus* sp.	*Abies alba*, *Castanea denatata*, *C. crenata*, *C. sativa*, *Prunus* sp.	*Fagus sylvatica*, *Quercus robur*, *Acer platanoides*, *Aesculus hippocastanum*, *Alnus glutinosa*, *Rhododendron* sp., *Pieris* sp.
***Phytophthora gonapodyides***	*Juglans regia*, *Malus domestica*, *Quercus* sp.	*Corylus avellana*, *Rhododendron* sp.	*Chamaecyparis lawsoniana*, *Fagus sylvatica*
***Phytophthora plurivora***		*Abies alba*, *Acer platanoides*, *Acer pseudoplatanus*, *Qercus petrea*, *Tilia cordata*, *Rhododendron* sp., *Syringa vulgaris*	*Aesculus hippocastanum*, *Alnus glutinosa*, *Fraxinus excelsior*, *Quercus robur*, *Fagus sylvatica*, *Carpinus betulus*
***Phytophthora quercina***			*Quercus* spp.
***Phytophthora cinnamomi***		*Abies* sp.	*Castanea sativa*, *Quercus* sp., *Chamaecyparis lawsoniana*
***Phytophthora citrophthora***	*Juglans regia*	*Buxus* sp., *Castanea sativa*	*Citrus* sp., *Aesculus hippocastanum*, *Chamaecyparis lawsoniana*, *Rhododendron* sp.
***Phytophthora cryptogea***	*Juglans regia*, *Malus domestica*, *Pinus mugo*, *Pinus nigra*, *Pinus contorta*	*Abies concolor*, *Abies fraseri*, *Abies procera*, *Cupressus* sp., *Pseudotsuga menziesii*	*Chamaecyparis* sp., *Rhododendron catawbiense*, *Rhododendron maximum*
***Phytophthora ramorum***		*Abies* sp., *Aesculus hippocastanum*, *Alnus* sp., *Betula pendula*, *Fagus sylvatica*, *Fraxinus excelsior*, *Quercus* sp.	*Notholithocarpus densiflorus*, *Rhododendron ponticum*, *Rhododendron* sp., *Larix kaempferi*, *Pseudotsuga menziesii*
***Phytophthora pseudosyringae***		*Quercus* spp., *Carpinus betulus*	*Aesculus hippocastanum*, *Fagus sylvatica*, *Alnus glutinosa*
***Phytophthora syringae***	*Prunus* sp.		*Aesculus hippocastanum*, *Fagus sylvatica*, *Camelia* sp., *Rhododendron* sp.
***Phytophthora rosacearum***			*Malus domestica*, *Prunus* spp.
